# Acting on sex and gender in medical innovation is good for business

**DOI:** 10.1136/bmj-2022-072242

**Published:** 2023-06-07

**Authors:** Lavanya Vijayasingham, Evelyne Bischof, Bernadette Ateghang-Awankem, Maryam Rumaney, Mariam Otmani del Barrio, Phaik Yeong Cheah, Ajoke Sobanjo ter-Meulen, Cara Tannenbaum, Rosemary Morgan, Jeannette Wolfe

**Affiliations:** 1 London School of Hygiene and Tropical Medicine, London, UK; 2 Shanghai University of Medicine and Health Sciences, College of Basic Medicine, Shanghai, China; 3 Renji University Hospital of Jiatong School of Medicine, Renji, Shanghai, China; 4 International Center for Multimorbidity and Complexity in Medicine (ICMC), Universität Zürich, Switzerland; 5 Global Health Division, Pan African Health Systems Network, Nussloch, Germany; 6 Cape Town, South Africa; 7 Unicef/UNDP/World Bank/WHO Special Programme for Research and Training in Tropical Diseases, World Health Organization, Geneva, Switzerland; 8 Mahidol-Oxford Tropical Medicine Research Unit (MORU), Faculty of Tropical Medicine, Mahidol University, Thailand; 9 Centre for Tropical Medicine and Global Health, Nuffield Department of Medicine, University of Oxford, Oxford, UK; 10 Department of Global Health, University of Washington, Seattle, USA; 11 Faculties of Medicine and Pharmacy at the Université de Montréal, Québec, Canada; 12 Institute of Gender and Health, Canadian Institutes of Health Research, Canada; 13 Department of International Health, Johns Hopkins Bloomberg School of Public Health, Baltimore, USA; 14 Department of Emergency Medicine, UMass Chan Medical School-Baystate Campus, Springfield, USA

## Abstract

**Lavanya Vijayasingham and colleagues** argue that as well as improving safety and efficacy, considering sex and gender related factors in medical research can have commercial benefits

Earlier and stronger attention to sex and gender factors during medical product research and development, including vaccines, was widely called for by sex and gender experts before the covid-19 pandemic.^1^ ^2^ A combination of biological sex and gender factors (box 1) are widely accepted to influence the safety, efficacy, and acceptability of products, which can ultimately influence the uptake of lifesaving and quality of life enhancing innovations.^1^ ^3^ ^4^ Policies to promote the integration of sex and gender in health research have been implemented by multiple research funders, evaluation and regulatory bodies, and influential journals.^1^ ^2^ ^5^ Yet those who funded, developed, and brought the covid-19 vaccines to market did not adequately consider sex and gender in clinical trial design and reporting; nor did they sufficiently act on the concerns and needs of women, who have been disproportionally affected by historical biases in medical research.

Box 1 **Definitions**

**Biological sex factors** relate to the genetic, cellular, biochemical, physiological, and immunological nuances within and beyond the reproductive system that may variably influence the way medicines and vaccines act and move through the body
**Gender factors** refer to the socially constructed norms, behaviours, roles, differences in access to resources, and power relations experienced and navigated by those who identify as men, women, or gender diverse.

Innovators, investors, and industry tend to use cost as an excuse for excluding sex and gender related factors—that the extra work and resources would increase research costs and raise the product price.^1^ ^6^ ^7^ Some examples of activities that add time, complexity, and cost to research include recruiting more people in clinical trials to show sex differentiated outcomes, analytical methods to account for hormonal changes in menstrual cycles, and navigating the risks, legal liabilities, and protectionary ethics of conducting research during pregnancy and lactation.^1^ ^6^ ^7^ ^8^

Existing and emerging technologies to support the overall therapeutics and vaccines development process, such as artificial intelligence and machine learning, can theoretically simplify and mitigate risks and cut costs associated with these activities to offer value and affordable innovation to consumers.^9^ ^10^ Hence, we present an idea that may align with industry’s and investors market oriented motivations: rectifying data gaps on sex and gender related factors could increase the demand for, use, and effectiveness of medical and vaccine research, which would also serve commercial and market oriented goals.

## Sex and gender gaps in covid-19 vaccine research

Pregnancy was quickly identified as increasing the risk of severe covid-19 and related complications.^11^ The World Health Organization stated that pregnant women should not be routinely excluded from research participation.^12^ Yet, pregnancy was an exclusion criterion in initial phase 3 clinical trials, and when the first covid-19 vaccines were authorised for emergency use in late 2020, there were no formal clinical trial data on pregnancy, though developmental and reproductive toxicity studies were in progress.^13^ In the absence of formal clinical trial data, global health authorities assessments and recommendations were based on real time analysis of real world data. This led to the public perceiving mixed messages from global and national health agencies such as WHO and the US Centers for Disease Control and Prevention, and vaguely worded advice on the use of these vaccines during pregnancy from national professional societies.^14^ For pregnant women and people with early access to covid vaccines, especially pregnant frontline health workers, personal vaccination decisions were guided by advice from their healthcare professionals, permissive recommendations from mainly high income countries, and real time tracking of real world outcomes.^13^ ^14^ ^15^ ^16^ ^17^

Despite known sex and gender related differences in respiratory infections and in the safety and efficacy of vaccines^2^ ^18^ the supporting clinical trial reports for the regulatory evaluation process to facilitate the emergency use of covid-19 vaccines largely omitted sex disaggregated safety data, although sex disaggregated efficacy data were provided to some major regulatory agencies (US Food and Drug Administration, European Medicines Authority, Health Canada).^19^ Published clinical trial reports rarely included both sex disaggregated safety and efficacy outcomes.^20^ ^21^

Some evidence of sex and gender related differences emerged once the vaccines were used outside trials. When vaccines began to be available to priority populations (healthcare workers) in early 2021, there were early reports of rare allergic reactions to mRNA vaccines and of thrombosis associated with viral vector vaccines, especially in women.^7^ ^22^ Early post-vaccination survey reports from the UK and US also suggested that females reported more non-serious side effects than males.^23^ ^24^ Serious adverse events such as myocarditis and pericarditis linked to the use of mRNA vaccines were higher in males, particularly in adolescent and adult males (aged 12-40 years).^25^

There were also global reports of temporary menstrual changes and irregularities soon after covid-19 vaccinations, including heavier bleeding, longer and more frequent periods, missed periods, and breakthrough bleeding in those who do not normally menstruate.^26^ ^27^ ^28^ Menstrual changes have also been reported after human papillomavirus vaccinations^29^ and viral infections are also known to induce menstrual changes in some instances.^29^ ^30^ In October 2022, the European Medicines Authority Pharmacovigilance Risk Assessment Committee recommended that heavy menstrual bleeding be added as a “side effect of unknown frequency” related to mRNA covid-19 vaccines.^31^

## Missed opportunities to influence uptake 

Inadequate attention to sex and gender related factors in covid-19 vaccine research led to missed opportunities to counter preconceptions and unresolved concerns about the safety of covid-19 vaccines, which may have negatively influenced the equitable and timely uptake. Early research conducted in 2020 in the UK and US suggested that more women than men were hesitant about covid-19 vaccine safety and side effects.^32^ ^33^ Global studies on vaccine acceptance, including those from low and middle income countries, also found higher vaccine acceptance in men than women.^34^ ^35^ A survey of 1181 pregnant women conducted during August to October 2020 in the UK found that their acceptance of the covid-19 vaccine while pregnant was much lower than when not (62% *v* 81%).^36^ A global survey of 17 871 women across 16 countries similarly found a lower willingness to be vaccinated among those who were pregnant than among those who were not (52% (2747/5282) *v* 73.4% (9214/12 562)), but these proportions varied across countries.^37^ The main issues that shaped negative responses in both surveys were concerns about safety and the vaccine development process, including the speed of development, the lack of data, and potential adverse or long term effects.^36^ ^37^ These findings signal the need to engage and communicate with diverse groups of women to resolve their concerns.

Communication campaigns were developed to counter myths and misinformation that the covid-19 vaccine could affect fertility,^38^ and research has since confirmed that male and female fertility is not affected by covid-19 vaccination.^39^ Similarly, nuanced engagement, research, and communication targeting diverse groups of women might have helped anticipate and address women’s concerns, perspectives, and needs, particularly among those sceptical about vaccine safety or who linked menstrual changes with fertility issues.^26^ ^36^ ^37^ ^38^

## Realising the market oriented potential of sex and gender 

Science that acts on sex and gender related factors in research and development of medicines and vaccines can fill some of the data and communication gaps outlined, which then can increase the demand for, use, and impact of research as well as helping to meet commercial and market oriented goals. We offer some strategies as a starting point for industry, innovators, and investors.

### Explore female centric market dynamics and build a socially conscious brand 

Countering gaps in sex related data and other dynamics of gender inequality can be good for business. Companies that work to address these issues can be perceived as socially conscious and responsive to user needs and preferences by target consumers, investors, and health payers.^7^ For example, the market for “femtech”—health technologies such as wearable devices and phone apps to monitor menstruation and fertility, discreet and comfortable breast pumps, and menstrual health products—is expected to grow to about $1tn by 2026.^40^ Femtech products currently focus on reproductive health needs but show the commercial value of better understanding of and filling women’s health needs, including through intentional focus on their concerns in the development of therapeutics and vaccines.

### Listen to and heed women’s voices 

Engagement and involvement activities can also enhance market advantage and value creation processes. For example, the meaningful involvement of diverse groups of women throughout the research and development process enables their perspectives and needs to be better understood and addressed.^41^ Engagement can range from input on study designs to ensure relevant outcomes and trial recruitment and retention strategies, through to dialogue on product information materials and marketing strategies.^42^ ^43^ Market research on the concerns and acceptability of forthcoming products can extend into active co-design of therapeutic product profiles, service, or communication strategies that better reflect women’s experiences of illness, health preferences, decision making patterns, and care challenges. Resources such as the WHO R&D Blueprint good participatory practice toolbox provide approaches to engage communities, and apply participatory processes, including in trial participation, adherence, and retention.^44^ ^45^

### Routinely include pregnant and breastfeeding women in trials

If value is derived from filling unmet needs, then there is a huge market gap in using research to protect pregnant women and their unborn babies from the harm of serious health conditions, rather than solely protecting them from the unanticipated harms of research on a potentially life saving intervention. Industry, innovators, and investors must continue to engage with and co-develop strategies with regulators and expert scientists to mitigate risks and uncertainties that have constrained the inclusion of women who are pregnant or breastfeeding in clinical trials. Lessons from covid-19 and the Zika epidemic published by the Covax Maternal Immunisation Working Group^13^ and the Pregnancy Research Ethics for Vaccines, Epidemics, and New Technologies (PREVENT) Working Group^46^ should guide vaccine development in future disease outbreaks. 

### Consider men, non-binary, and non-cis gender populations 

Although historical gaps in research tend to disadvantage women, there is also value in ensuring no other gender groups are similarly left behind in future medical research. This includes non-binary and gender diverse groups as well as men when there is an unmet need. Movement away from a binary, cisgender conceptualisation of sex and gender can support research into if and how the safety and efficacy profiles differ across sex and gender identity groups. As an example, changes in the body physiology, composition, and biochemistry of transgender people who have gender affirming treatments such as surgery or long term hormone therapy (oestrogen or testosterone) may mean they respond to drugs differently from cisgender women and men.^47^ ^48^

### Report sex disaggregated data simply

Reporting of sex disaggregated data can be used as a science communication strategy that can influence women’s health related decision making. Research data are no longer just required for regulatory approvals. Data narratives shape public perceptions and demand for a product, as shown by women’s perceptions of the new covid-19 vaccines. Nuanced reporting, with attention to known concerns—for example, from experiences of previous epidemics or vaccination programmes—can better support women and other gender identity groups as health consumers to make informed decisions about their health. Indeed, ambiguous, small sample size, or non-clinically meaningful sex differences are often intentionally not reported in research journals to avoid misinterpretations and potential misuse of the information in creating inequitable access policies or uptake decisions.^49^ ^50^

During the pandemic, preprints and covid-19 related journals were publicly accessible. In some cases this resulted in premature media coverage and inaccurate public understanding of research findings.^51^ These problems can be partially avoided by including a clear description of the limitations and interpretation of the results, alongside sex disaggregated data. This information can also inform the design of future trials or analytical synthesis to further verify and document the influence of sex related factors.^50^

## Inclusive innovation requires inclusive collaboration

Acknowledging that industry, innovators, and investors value market oriented outcomes, we contend that filling data and communications gaps on sex and gender related factors in medicines and vaccines can be good for the advancement of medical science, which for commercial organisations is also good for business. Of course, commercial gains should not be the only reason to motivate change. Stronger action on sex and gender related factors also promotes safer and more precise science, as well as more equitable, inclusive, and human rights driven innovation.^7^ All these reasons have been articulated many times by gender experts. 

To foster and accelerate change, regulators must continue to implement and enforce sex and gender related policies to normalise practice, including through dialogue with industry, innovators, and investors, and providing conditions that will support consistent action on sex and gender factors.^1^ ^7^ Regulatory or policy levers are unlikely to be effective without support from industry and funders. Existing gender experts, advocates, and allies can collaborate more closely with industry, innovators, and investors to tackle sex and gender factors in meaningful ways, while also holding them accountable for their actions. We are optimistic that lessons from the covid-19 pandemic will shift mindsets to expand the global pool of gender champions who stimulate stronger action on sex and gender related factors in medical innovation.

**Key messages**

Insufficient consideration, analysis, and reporting of sex and gender related factors in medical research and development continues to disadvantage women and other gender groups
The omission of pregnant women from early covid-19 vaccine research made decisions about safety difficult and affected uptake
Failure to study sex and gender related factors can lead to missed opportunities to prevent morbidity and mortality, build trust, counter misinformation, and increase market size
Industry, innovators, and investors can benefit from including sex and gender related factors in all product development strategies

## Notes

Contributors and sources: The authorship team has expertise and experience in, health gender, clinical research, epidemiology, bioethics, internal medicine, emergency medicine, critical care, geriatrics, paediatrics, psychiatry, basic, translational, and clinical research, including vaccine development, laboratory science, health systems and public health research within low, middle, and high income countries. Their careers span work in academia, aid organisations, private for-profit, the pharmaceutical industry, tertiary hospitals, civil society, and the United Nations system. LV was part of the core United Nations University-International Institute for Global Health and University of Western Cape Gender and COVID-19 Research Agenda Setting Initiative research team, and led the article content development and writing process. JW, EB, BA-A, and RM acted as co-leads for thematic discussions, and MR was a coordinator. All authors contributed to the content, writing, and revisions of this article. LV is the guarantor. The views expressed do not necessarily represent the views, decisions, or policies of the institutions with which the authors are affiliated.

Provenance and peer review: Commissioned; externally peer reviewed.

This article is part of a collection proposed by the United Nations University and the University of the Western Cape and commissioned by *The BMJ*. *The BMJ* peer reviewed, edited, and made the decision to publish these articles. Article handling fees are funded by Bill and Melinda Gates Foundation and UN University-International Institute for Global Health.
